# Manifestation of depression in speech overlaps with characteristics used to represent and recognize speaker identity

**DOI:** 10.1038/s41598-023-35184-7

**Published:** 2023-07-10

**Authors:** Sri Harsha Dumpala, Katerina Dikaios, Sebastian Rodriguez, Ross Langley, Sheri Rempel, Rudolf Uher, Sageev Oore

**Affiliations:** 1 Faculty of Computer Science, Dalhousie University, Halifax, NS Canada; 2 Vector Institute, Toronto, ON Canada; 3 Dalhousie University, Psychiatry, Halifax, NS Canada; 4 Nova Scotia Health, Halifax, NS Canada

**Keywords:** Biomarkers, Computer science

## Abstract

The sound of a person’s voice is commonly used to identify the speaker. The sound of speech is also starting to be used to detect medical conditions, such as depression. It is not known whether the manifestations of depression in speech overlap with those used to identify the speaker. In this paper, we test the hypothesis that the representations of personal identity in speech, known as speaker embeddings, improve the detection of depression and estimation of depressive symptoms severity. We further examine whether changes in depression severity interfere with the recognition of speaker’s identity. We extract speaker embeddings from models pre-trained on a large sample of speakers from the general population without information on depression diagnosis. We test these speaker embeddings for severity estimation in independent datasets consisting of clinical interviews (DAIC-WOZ), spontaneous speech (VocalMind), and longitudinal data (VocalMind). We also use the severity estimates to predict presence of depression. Speaker embeddings, combined with established acoustic features (OpenSMILE), predicted severity with root mean square error (RMSE) values of 6.01 and 6.28 in DAIC-WOZ and VocalMind datasets, respectively, lower than acoustic features alone or speaker embeddings alone. When used to detect depression, speaker embeddings showed higher balanced accuracy (BAc) and surpassed previous state-of-the-art performance in depression detection from speech, with BAc values of 66% and 64% in DAIC-WOZ and VocalMind datasets, respectively. Results from a subset of participants with repeated speech samples show that the speaker identification is affected by changes in depression severity. These results suggest that depression overlaps with personal identity in the acoustic space. While speaker embeddings improve depression detection and severity estimation, deterioration or improvement in mood may interfere with speaker verification.

## Introduction

Major depressive disorder, also known as depression, is a common mental disorder and a leading cause of disability worldwide^1^. According to the World Health Organization^2^, more than 300 million people (around $5\%$ of the global population) are living with depression. Early and objective diagnosis of depressive symptoms is crucial in reducing the burden of depression, but inadequate access to clinical services and associated stigma limit detection. In addition to depression identification, it is important to measure the severity of depression as repeated measurements are needed to guide effective treatment and improve outcomes^3^. Measurement-based care is known to be effective, yet it is underused in practise because of the perceived burden of existing measurement tools^4^. For treatment purposes, automated assessment systems would have potential to help, if they could detect and measure depression with some reliability from easy-to-obtain material. Automated assessment systems may facilitate the detection and treatment of depression if they could reliably detect and measure depression in easy to obtain material.

Audio recording of speech is easy to obtain and may contain sufficient information for the detection and measurement of depression^5–7^. The potential vocal biomarkers for depression explored in previous works include a range of acoustic features, such as prosodic characteristics (e.g., pitch and speech rate), spectral characteristics (e.g., Mel-frequency cepstral coefficients and formant frequencies), and glottal (vocal fold) excitation patterns^8–11^. Previous work explored spectral, prosodic and glottal features for depression detection and severity estimation, but the accuracy and generalizability of depression detection is limited by the size of samples with available diagnostic information. Obtaining large samples of speech with diagnostic information is expensive and associated with ethical challenges of datasets combining identifiable (voice) and sensitive (diagnosis) information. One way of making better use of valuable datasets of limited size is to use models pre-trained on different but related tasks in much larger datasets.

Speech audio is routinely used for recognizing the identity of the speaker. Voice-based speaker identification is highly accurate thanks to models trained on large corpus; for instance the VoxCeleb2^12^ dataset includes 3000 hours of speech by 7160 speakers. The experience of depression is intimately connected with the core of a person’s identity^13^. Depression is associated with self-focused attention and altered perception of the self^14^. The change between depressed and well states is so striking that recovery is commonly described as being a ’different person’. Based on the intimate link between depression and personal identity, we hypothesized that a model pre-trained for speaker identification will improve the detection of depression and estimation of depression severity from natural speech. In this work, we test this hypothesis by exploiting the representations of personal identity, known as speaker embeddings, in the detection and measurement of depression in speech.

To qualify the above hypothesis, we define speaker embeddings as text-independent speaker-specific information that include acoustic characteristics that are independent of what the speaker is saying. Speaker embeddings represent not only the identifiable information such as gender, age, etc., but have been shown to provide important cues about the traits of the speaker such as personality, physical state, likability, and pathology^15^. Speaker embeddings extracted from speech have previously been used for tasks such as automatic speaker verification^16^, improving speech recognition performance^17^, multi-speaker speech synthesis^18^, and emotion classification^19^. In this work, we apply speaker embeddings to the tasks of depression detection and severity estimation from speech. We empirically show that the speaker characteristics of an individual—as represented by speaker embeddings—are affected by changes in depression severity of the individual. We consider three established variants of speaker embeddings; the x-vectors, ECAPA-TDNN (Emphasized Channel Attention, Propagation, and Aggregation Time-delay neural network) x-vectors^20^, and d-vectors^21^. By using speaker embeddings, we demonstrate that large, public, unlabeled datasets *in conjunction with* much smaller labeled datasets, can be leveraged to improve on the state-of-the-art (SOTA) performance in clinically meaningful tasks with implications for public health.

**Figure 1:**
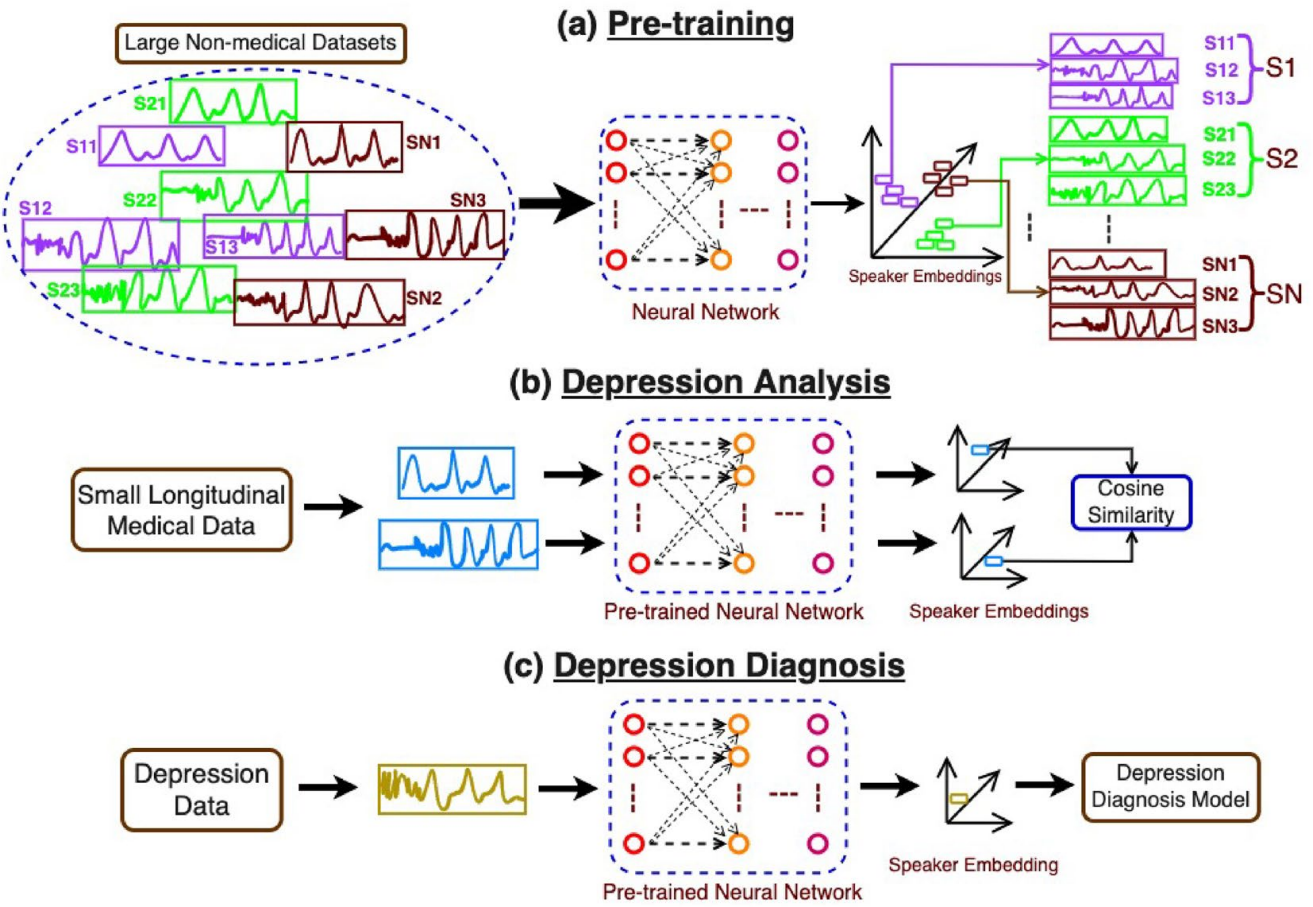
Schematic depiction of the outline of the paper. There are three different phases in this work (**a**) Pre-training for speaker embeddings using a large non-medical speech data collected from *N* different speakers, (**b**) Depression analysis using speaker embeddings extracted from pre-trained models on longitudinal data, and (**c**) Depression detection and severity estimation using speaker embeddings extracted from pre-trained models.

### Related work

The application of deep learning techniques significantly boosted the performance of depression detection using speech^22–27^. Initial work on speech-based depression detection used deep neural networks (DNNs) with fully-connected layers^22^. Then, convolutional neural networks (CNNs) and recurrent neural networks with long short-term memory (LSTM) units achieved better performance for depression detection and severity estimation^23,24^. Later, CNN-LSTM, dilated CNN and dilated CNN-LSTM models improved the SOTA performance in depression detection and severity estimation^25–28^. Further, sentiment and emotion embeddings were used for depression severity estimation^29^. To the best of our knowledge, none of the previous studies have explored the application of speaker embeddings for depression detection and severity estimation. i-vector-based models have been trained from scratch for detecting depression^30–32^, but these studies did not use i-vector models to extract speaker embeddings for depression detection. In this work, we use speaker embeddings to train multi-kernel CNN (MK-CNN)^33^ and LSTM models for depression detection and severity estimation.

## Methods

Our method consists of three phases, (1) Pre-training, (2) Depression analysis on longitudinal data, and (3) Depression detection and severity estimation. In pre-training phase of the speaker embedding models, given speech data collected from a large pool of speakers, we train speaker classification models to classify the speech samples based on the speaker labels. In the second phase, we use longitudinal data to analyze the effect of the changes in depression severity on speaker embeddings of an individual. In the third phase, we analyze the significance of speaker embeddings for the task of depression detection and severity estimation using speech. We use the speaker embeddings extracted using the pre-trained speaker classification models (trained in the first phase) in the second and third phases. Figure 1 shows an overview of our method.

**Table 1:** Details of the DAIC-WOZ and Vocal Mind datasets.

Metrics	DAIC-WOZ	Vocal Mind
	Dataset	Dataset
Data collection format	Interview	Spontaneous
Severity rating scale	PHQ-8	MADRS
Total samples	219	514
Total duration (in h)	59	41
Total participant speech duration (in h)	32	37
	Count (%)	Count (%)
Female speakers	92 (42%)	390 (76%)
Male speakers	127 (58%)	124 (24%)
Non-depressed samples	154 (70%)	403 (78%)
Depressed samples	65 (30%)	111 (22%)
	Mean (Std.)	Mean (Std.)
Sample duration (in min)	16.04 (4.55)	4.79 (1.04)
Participant speech Duration (in min)	8.75 (5.02)	4.33 (1.09)
Age	40.70 (12.53)	43.58 (16.97)
Severity score	6.64 (6.01)	6.41 (6.05)

Std. refer to standard deviation.

### Dataset

In this work, we used two depression datasets, DAIC-WOZ^34^ ((Distress Analysis Interview Corpus - Wizard of Oz—a corpus of clinical interviews) and Vocal Mind (spontaneous speech corpus obtained in a clinical setting) for analysis. DAIC-WOZ dataset contains a set of 219 clinical interviews collected from 219 participants (154 healthy and 65 depressed). Each audio sample was labeled with a PHQ-8 (Patient Health Questionnaire) score, in the range of 0–24, to denote the severity of depression. Vocal Mind dataset contains speech samples collected from 514 participants (403 healthy and 111 depressed). Depression severity of each speech sample was scored on the Montgomery and Asberg Depression Rating Scale (MADRS), which is in the range of 0–60. Further, longitudinal speech data also collected as a part of the Vocal Mind project was used. Longitudinal speech data was collected from 65 individuals at different dates, where variations in their depression severity scores were observed during this period. Manual transcripts with timestamps of the DAIC-WOZ and Vocal Mind datasets were used to discard the interviewer speech segments and retain only the participant speech segments for analysis. The retained participant speech segments were combined and were then divided into non-overlapping segments of 5–6 seconds in duration. This resulted in 15710 and 25144 segments for DAIC-WOZ and Vocal Mind datasets, respectively. The depression label assigned for each segment is same as the label of the entire speech sample. For DAIC-WOZ dataset, speech samples with PHQ-8 scores greater than or equal to 10 (PHQ-8 $\ge$ 10) were considered as depressed and those samples with PHQ-8 scores less than 10 (PHQ-8 < 10) were considered as healthy. This corresponds to the recommended threshold for depression identification^35,36^. For the Vocal Mind dataset, speech samples with MADRS greater than or equal to 10 (MADRS $\ge$ 10) were considered as depressed and those samples with MADRS less than 10 (MADRS < 10) were considered as healthy. This corresponds to the established threshold for remission on MADRS^37^. Table 1 provides various statistics of the DAIC-WOZ and the Vocal Mind datasets.

### Pre-training

We use the pre-trained models available in speech-brain^38^ for extracting the x-vectors and ECAPA-TDNN x-vectors from the speech samples. To extract d-vectors, we pre-trained the GE2E network on the task of speaker verification by consolidating *two large non-clinical datasets* (LibriSpeech^39^ and VoxCeleb2^12^), which are *publicly available*. LibriSpeech dataset consists of speech samples collected from 1166 speakers, and the VoxCeleb dataset consists of speech samples collected from 1166 speakers. In this work, *We did not fine-tune the pre-trained speaker classification models on the depression datasets (i.e., DAIC-WOZ and Vocal Mind datasets)*.

We then used these pre-trained models to extract speaker embeddings (x-vector, ECAPA-TDNN x-vectors, and d-vectors) at segment-level for the depression datasets. The dimensions of the speaker embeddings are 512, 256, and 192 for x-vector, ECAPA-TDNN x-vector, and d-vector, respectively. Finally, we use these speaker embeddings to train and test the LSTM and MK-CNN models for depression detection and severity estimation. We train separate models for x-vector, ECAPA-TDNN x-vector, and d-vector speaker embeddings.

### Speaker embeddings for depression

**Figure 2:**
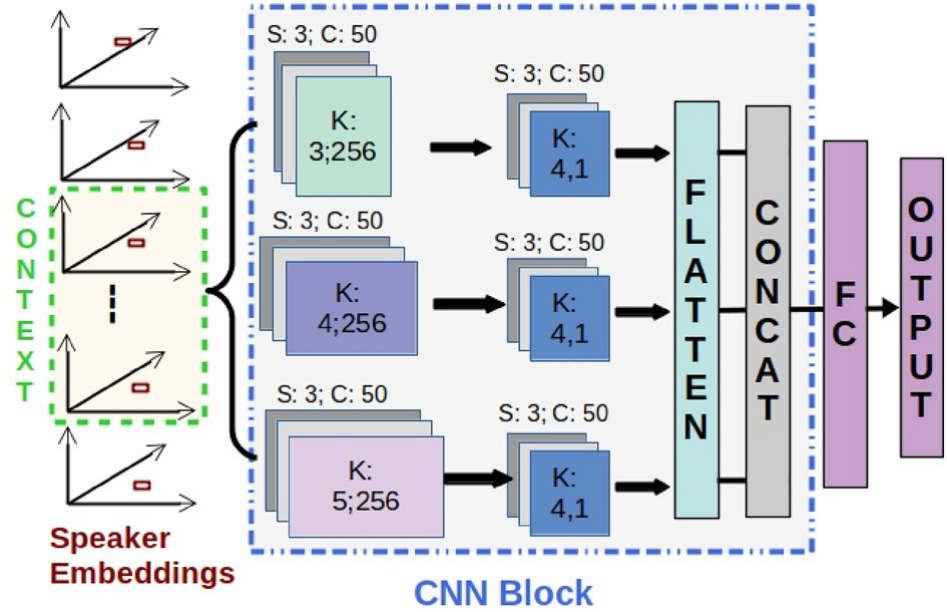
Network for depression detection using speaker embeddings as input. S, C, K refers to the stride, number of channels and kernel size of the convolutional layer, respectively. FC refers to a fully-connected layer. The same network is used for OpenSMILE and COVAREP features.

We train MK-CNN (shown in Fig. 2) and LSTM networks with different speaker embeddings for depression detection and severity estimation.

### MK-CNN model

We trained a MK-CNN model, as shown in Fig. 2, for depression detection and severity estimation using the extracted speaker embeddings. The first convolutional layer consists of 3 different kernels with sizes (3, *L*), (4, *L*), and (5, *L*), respectively. Here, *L* refers to the length of the input feature vector. *L* = 512, 256 and 192 for x-vector, ECAPA-TDNN x-vector and d-vector, respectively. Each kernel consists of 50 channels. In the second convolutional layer, the size of all kernels is 4, with 50 channels in each kernel. Outputs from each kernel of the second convolutional layer are flattened and then concatenated before passing through a fully-connected (FC) layer with 100 units and an output layer.

### LSTM model

We also trained an LSTM network for depression detection and severity estimation using the extracted speaker embeddings. The LSTM network is the same as the MK-CNN network shown in Fig. 2, with the MK-CNN block replaced by an LSTM block consisting of 2 LSTM layers with 128 units each. The output of the LSTM block, for the last timestep, is passed through an FC layer with 100 units and an output layer.

### Baseline DNN

We considered a fully-connected deep neural network (DNN) as a baseline for comparison. This DNN has three hidden layers with 128, 64, and 128 ReLU units, respectively, followed by an output layer.

Further, we extracted COVAREP^24^ and OpenSMILE^40^ features for performance comparison with speaker embeddings. COVAREP and OpenSMILE features, obtained at the segment level, were used to train and test the MK-CNN, LSTM, and DNN networks. We extracted the 384-dimensional OpenSMILE features using the *IS*09 configuration. We obtained the 444-dimensional COVAREP by computing the higher-order statistics (mean, maximum, minimum, standard deviation, skew, and kurtosis). We calculated statistics on the frame-level COVAREP features.

### Combining embeddings (CE)

**Figure 3:**
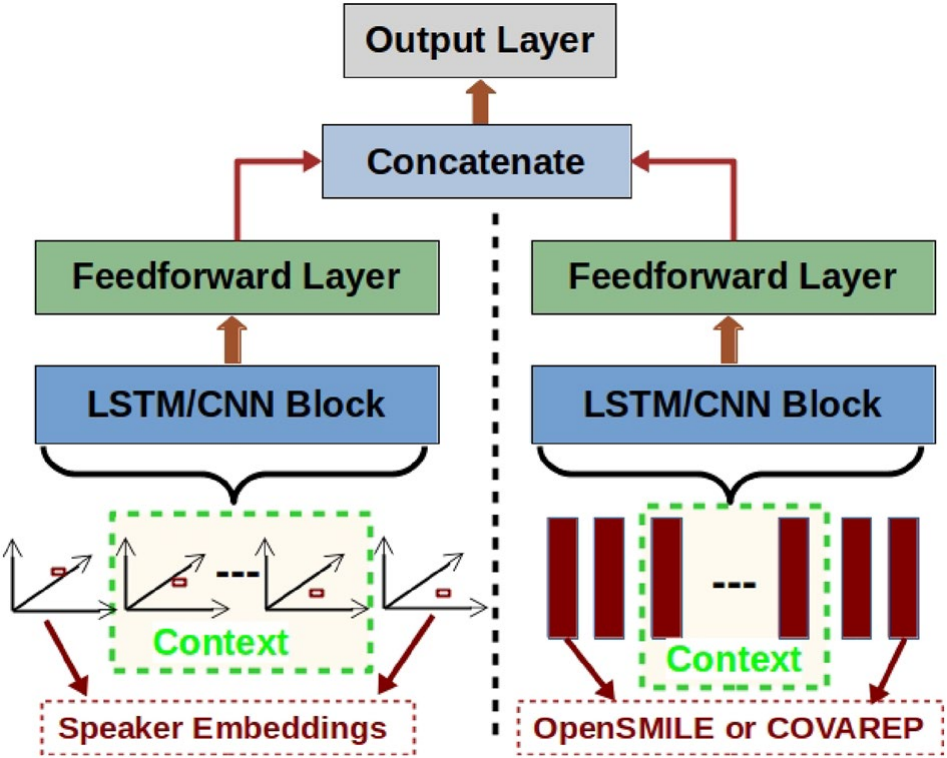
Network for combining speaker embeddings, and OpenSMILE or COVAREP features for depression detection.

We also try combining speaker embeddings (one of the x-vector, ECAPA-TDNN x-vector or d-vector) with the OpenSMILE or COVAREP features (as shown in Fig. 3), for depression detection and severity estimation. The proposed network consists of two branches, one for speaker embeddings and the other for OpenSMILE or COVAREP features. The input features to each branch are passed through an LSTM (CE$_{l}$) or MK-CNN (CE$_{c}$) block and then through a fully-connected (FC) layer (100 units). The outputs of the FC layer of each branch are combined using dot product and then passed through an output layer to get the final decision.

For all the above networks, the final output layer is a softmax with two units when trained for the task of depression detection and a single linear unit when trained for depression severity estimation. The context in Figs. 2 and 3 refers to the number of contiguous segments in an audio recording considered to train and test the models. We experiment with temporal contexts of different lengths to analyze the optimal number of contiguous speech segments required to train the models (see subsection ”Temporal Context in Depression Detection” in supplementary material). Even though the networks are trained and tested at segment-level with different contexts, the final performance metrics are obtained based on the prediction for the entire audio file. For depression detection, we use majority voting on the segment-level decisions for the final decision. For depression severity score prediction, we compute the mean of the segment-level scores to compute the overall depression severity score.

### Analysis of longitudinal data

Here, we performed experiments on longitudinal speech data to analyze whether the speaker embeddings of an individual change as the depression severity score of that individual varies. For this analysis, we used the longitudinal data collected from speakers. For the given longitudinal speech samples, we extracted and analyzed different speaker embeddings i.e., x-vector, , ECAPA-TDNN x-vector, and d-vector. We then computed the cosine similarity scores between the speaker embeddings of the longitudinal speech samples. We also noted the difference in MADRS scores between the longitudinal samples. Finally, we analyzed the cosine similarity (A.B = ||A|| ||B|| cos$\theta$) scores in relation to the variations in the MADRS score.

### Training details

We used Adam optimizer ($\beta _1=0.9$, $\beta _2=0.99$), with an initial learning rate of 0.0005, to train all the networks. Dropout rates of 0.3, 0.4, and 0.3 were used for the MK-CNN block, LSTM block, and FC layers, respectively. ReLU activation was used for all the CNN, LSTM, and FC layers. All networks were trained for 50 epochs using a batch size of 128. For training the depression detection model, we used the negative log-likelihood loss function. Whereas for training the depression severity estimation model, we used the mean-squared error loss function. Class weights were set based on the distribution of samples in the train set to alleviate the class imbalance issue during training. We maintained a constant value for temporal context (number of contiguous segments in a sample) across the train, validation, and test phases.

### Measurements

Depression detection performance is measured using the $F_1$ score ($F_1(D)$ and $F_1(H)$) and balanced accuracy (BAc.). $F_1(D)$ and $F_1(H)$ are the $F_1$ scores of depressed and healthy classes, respectively. Depression severity estimation performance is measured using root mean squared error (RMSE). The higher the $F_1$ and BAc. values, the better the performance. Similarly, the lower the RMSE values, the better the performance. We report results using 5-fold cross-validation. There is no speaker overlap between folds, and we maintain the same proportion of depressed and healthy participants across all the folds.

## Experiments and discussion

### Depression detection and severity estimation

Tables 2–4 provide the experimental results obtained using ECAPA-TDNN x-vector (ECAPA) based speaker embeddings. Table 2 shows the depression detection and severity estimation performance when ECAPA speaker embeddings are combined with the OpenSMILE ((ECAPA, OpenSMILE)) or COVAREP ((ECAPA, COVAREP)) features, respectively. Models trained on speaker embeddings outperformed the models trained on COVAREP or OpenSMILE features for DAIC-WOZ and Vocal Mind datasets. The depression detection and severity estimation performance further improved when the speaker embeddings were used in conjunction with the OpenSMILE or COVAREP features. This shows that the speaker embeddings and the OpenSMILE or COVAREP features carry complementary information. The performance of the LSTM models was better or comparable to the MK-CNN models. To obtain the results in Tables 2–4, we used a context of 16 segments for DAIC-WOZ, and a context of 20 segments for Vocal Mind datasets to train the LSTM and MK-CNN models. (see Supplementary Table S1 and S2 for the depression assessment results using x-vector and d-vector based speaker embeddings.)

**Table 2:** Depression detection and severity estimation performance, in terms of $F_1$ ($F_1(D)$ and $F_1(H)$), Balanced Accuracy (BAc.) and RMSE, on DAIC-WOZ and Vocal Mind datasets.

		Acoustic features Alone				Speaker embeddings Alone				Acoustic and speaker Embeddings combined			
Dataset1: DAIC	Model	COVAREP				ECAPA				(ECAPA, COVAREP)			
		$F_1(D)$	$F_1(H)$	BAc.	RMSE	$F_1(D)$	$F_1(H)$	BAc.	RMSE	$F_1(D)$	$F_1(H)$	BAc.	RMSE
	MK-CNN	0.35	0.70	0.52	7.39	0.43	0.78	0.60	6.35	0.45	0.79	0.61	6.21
	LSTM	0.32	0.70	0.51	7.41	0.46	0.79	0.61	6.31	**0.47**	**0.80**	**0.63**	**6.19**
		OpenSMILE				ECAPA				(ECAPA, OpenSMILE)			
	MK-CNN	0.37	0.74	0.55	6.87	0.43	0.78	0.61	6.35	0.49	0.81	0.65	6.08
	LSTM	0.39	0.73	0.56	6.82	0.46	0.79	0.63	6.31	**0.50**	**0.83**	**0.66**	**6.01**
Dataset2: VM	Model	COVAREP				ECAPA				(ECAPA, COVAREP)			
		$F_1(D)$	$F_1(H)$	BAc.	RMSE	$F_1(D)$	$F_1(H)$	BAc.	RMSE	$F_1(D)$	$F_1(H)$	BAc.	RMSE
	MK-CNN	0.30	0.68	0.49	7.61	0.32	0.80	0.55	6.64	0.34	0.80	0.57	6.55
	LSTM	0.32	0.67	0.50	7.63	0.34	0.81	0.57	6.62	**0.37**	**0.81**	**0.60**	**6.51**
		OpenSMILE				ECAPA				(ECAPA, OpenSMILE)			
	MK-CNN	0.32	0.74	0.53	6.96	0.32	0.80	0.56	6.64	0.41	0.81	0.61	6.41
	LSTM	0.34	0.75	0.54	6.94	0.34	0.81	0.57	6.62	**0.43**	**0.84**	**0.64**	**6.28**

$F_1(D)$ and $F_1(H)$ are $F_1$ scores for depressed and healthy classes, respectively. COVAREP and OpenSMILE are acoustic features. Results obtained using ECAPA-TDNN x-vectors (ECAPA), COVAREP and OpenSMILE features on DAIC-WOZ (DAIC) and Vocal Mind (VM) datasets. For results obtained by combining Acoustic and Speaker embeddings ((ECAPA, COVAREP) and (ECAPA, OpenSMILE)), MK-CNN and LSTM models refer to CE models with MK-CNN and LSTM blocks, respectively.

Bold values indicate best results in each comparison group.

**Table 3:** Performance comparison of proposed approach with SOTA approaches. CE_l_ refers to models with LSTM block.

	DAIC-WOZ				Vocal mind			
Approach	$F_1(D)$	$F_1(H)$	BAc.	RMSE	$F_1(D)$	$F_1(H)$	BAc.	RMSE
Sequence	0.32	0.70	0.51	7.41	0.32	0.67	0.49	7.63
eGeMAPS	0.32	0.71	0.52	7.05	0.27	0.74	0.50	7.22
FVTC-MFCC	0.37	0.79	0.58	6.41	0.30	0.77	0.54	6.85
FVTC-FMT	0.39	0.79	0.59	6.37	0.34	0.76	0.55	6.82
Mk-CNN (COVAREP)	0.35	0.70	0.52	7.39	0.30	0.68	0.49	7.61
LSTM (OpenSMILE)	0.39	0.73	0.56	6.82	0.34	0.75	0.55	6.94
MK-CNN (ECAPA-TDNN)	0.43	0.78	0.60	6.35	0.32	0.80	0.56	6.64
LSTM (ECAPA-TDNN)	0.46	0.79	0.63	6.31	0.34	0.81	0.57	6.62
CE$_l$ (ECAPA, COVAREP)	0.47	0.80	0.64	6.19	0.37	0.81	0.59	6.51
CE$_l$ (ECAPA, OpenSMILE)	**0.51**	**0.83**	**0.66**	**6.01**	**0.43**	**0.84**	**0.64**	**6.28**

**Table 4:** Performance comparison of the speaker embeddings with other pre-trained embeddings.

Model	DAIC-WOZ				Vocal Mind			
	$F_1(D)$	$F_1(H)$	BAc.	RMSE	$F_1(D)$	$F_1(H)$	BAc.	RMSE
Mockingjay	0.27	0.70	0.49	7.09	0.27	0.70	0.48	7.58
vq-wav2vec	0.32	0.71	0.52	6.95	0.25	0.73	0.49	7.12
wav2vec-2.0	0.38	0.74	0.55	6.77	0.32	0.74	0.52	7.03
TRILL	0.36	0.77	0.56	6.46	0.34	0.76	0.55	6.80
ECAPA (Proposed)	**0.46**	**0.79**	**0.63**	**6.31**	**0.34**	**0.81**	**0.57**	**6.62**

We compared the performance of our proposed approach with previous SOTA approaches for depression detection and severity estimation (see Table 3). In Sequence^24^, LSTM models trained with COVAREP features were used for depression detection and severity estimation. In eGeMAPS^41^, CNN models were trained using OpenSMILE features for depression detection. In FVTC-MFCC^27^, channel-delayed correlations of MFCCs were used to train dilated CNN models. In FVTC-FMT^27^, channel-delayed correlations of formant frequencies were used to train dilated CNN models. None of these approaches explicitly considered speaker-specific features for depression detection. Table 3 shows that the models trained on speaker embeddings performed better (or at least comparable to) than the SOTA approaches for speech-based depression detection and severity estimation tasks. The depression detection and severity estimation performances obtained by combining speaker embeddings with the OpenSMILE features ((ECAPA, OS)) outperformed the previous SOTA approaches.

**Figure 4:**
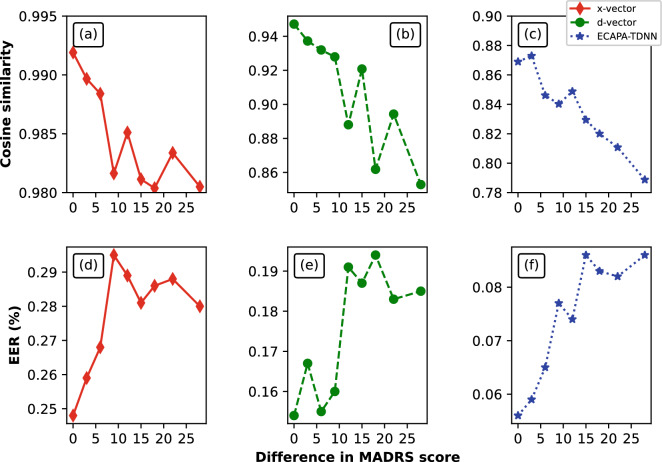
Analysis of speaker embeddings with respect to changes in depression severity scores using longitudinal data. (**a**–**c**) shows the variation in cosine similarity scores (between speaker embeddings extracted from longitudinal data) when the difference in MADRS score changes. (**d**–**e**) shows the variation in equal error rates (EER) (for the task of speaker classification) with respect to the difference in MADRS score between longitudinal samples. The different speaker embeddings are x-vector, d-vector and ECAPA-TDNN x-vector.

### Estimating depression from demographic variables

To understand the extent to which speaker embeddings make use of information beyond demographics such as biological sex and age for depression assessment, we trained machine learning models (decision trees, support vector machines and DNNs) for depression detection and severity estimation when only biological sex and age are provided as input. We found that the best performance obtained on the Vocal Mind dataset by combining biological sex and age ($F_1(D)$ = 0.16, $F_1(H)$ = 0.65 and GM = 0.32, RMSE = 8.35) was significantly worse than the performance obtained by the speaker embedding ($F_1(D)$ = 0.34, $F_1(H)$ = 0.81 and GM = 0.55, RMSE = 6.62). This shows that the speaker embeddings capture more information that is relevant for depression detection and severity estimation than just biological sex and age. Further details are provided in Supplementary Table S3.

Previous works reported that some machine learning models simply learned gender-specific information from the voice for depression detection^42–44^. To analyze the contribution of the gender-agnostic information contained in speaker embeddings for depression detection, we performed gender-specific depression detection as done in previous works^43,44^. We observed from the experimental results that the speaker embeddings do not rely completely on the gender-specific information for depression detection. For the DAIC-WOZ dataset (see Supplementary Table S4a), both Female and Male models achieved similar performance with the Female model performing slightly better than the Male model. Whereas for the Vocal Mind dataset (see Supplementary Table S4b), there is a large difference between the performance of the Female and the Male models, with the Female model performing significantly better than the Male model. but could this also be partially explained if, e.g. males depression does not manifest as clearly in their voice? or maybe that is the point here? This might be attributed to the difference in imbalance ratio between non-depressed to depressed samples in each gender: for females, the imbalance ratio between non-depressed to depressed = 294:95 $\approx$ 3:1 whereas for males the imbalance ratio between non-depressed to depressed = 109:16 $\approx$ 7:1. Experimental results are provided in Supplementary Table S4.

### Comparison with other pre-trained embeddings

We compared the performance of the proposed speaker embeddings (d-vector and ECAPA-TDNN x-vectors) with embeddings extracted using other pre-training techniques such as Mockingjay^45^, vq-wav2vec^46^, wav2vec 2.0^47^, and TRILL^48^. We trained the MK-CNN and LSTM networks with the speech-based embeddings extracted from the different pre-trained models. In Table 4, we reported results obtained using the LSTM networks (LSTM models performed better than the MK-CNN models across different embeddings). Speaker embeddings (both d-vector and ECAPA-TDNN x-vectors) performed better than the speech-based embeddings extracted using other pre-trained models. This signifies that the speaker embeddings alone could provide effective cues for detecting depression and estimating the severity of depression.

### Effect of depression on speaker embeddings in longitudinal data

Figure 4a–c shows the mean cosine similarity scores plotted with respect to the difference in MADRS scores between longitudinal speech samples. As the difference in the MADRS score increases, the cosine similarity value decreases. For longitudinal speech samples of a speaker, the higher the variation in MADRS score, the higher the variation in speaker embeddings for that speaker.

Figure 4d–f shows the mean equal error rates (EER in %) plotted with respect to the difference in MADRS scores between longitudinal speech samples. As the difference in the MADRS score increases, the EER values increases. This further confirms that for longitudinal speech samples of a speaker, the higher the variation in MADRS score, the higher the variation in speaker embeddings of that speaker.

It can also be observed that the variance or EER in speaker embeddings increase as the difference in depression severity scores increase. One reason for this behavior could be the skewed distribution of the samples across different values. There are more longitudinal samples with low differences in depression severity compared to samples with higher differences in depression severity. This might have led to higher variance at the end of the curve. Higher number of longitudinal samples might give us a better understanding of this behavior.

### Analysis of the speaker embeddings

We also analyzed the effectiveness of the extracted speaker embeddings (d-vector and ECAPA-TDNN x-vectors) for the task of speaker classification. The DAIC-WOZ dataset consists of recordings from 189-speakers—189-class speaker classification. Similarly, the Vocal Mind dataset consists of recordings from 514-speakers — 514-class speaker classification. We randomly selected 25 and 15 non-overlapping segments from each speaker to form the train and test sets for that speaker. We extracted ECAPA-TDNN x-vectors and d-vectors for all the samples. We trained logistic regression classifiers (with no hidden layers) separately on the d-vectors and ECAPA-TDNN x-vectors for the task of speaker classification. Speaker classification results are reported in terms of equal error rate (EER)—lower the value of EER, better the performance. Using d-vectors, we achieved EERs of 1.29 and 1.69 on the test sets of DAIC-WOZ and Vocal Mind datasets, respectively. Using ECAPA-TDNN x-vectors, we achieved EER values of 1.10 and 1.46 on the test sets of DAIC-WOZ and Vocal Mind datasets, respectively. These low EER values show that the extracted speaker embeddings carry crucial information about the speaker-specific characteristics.

### Comparison with a no-information system

**Figure 5:**
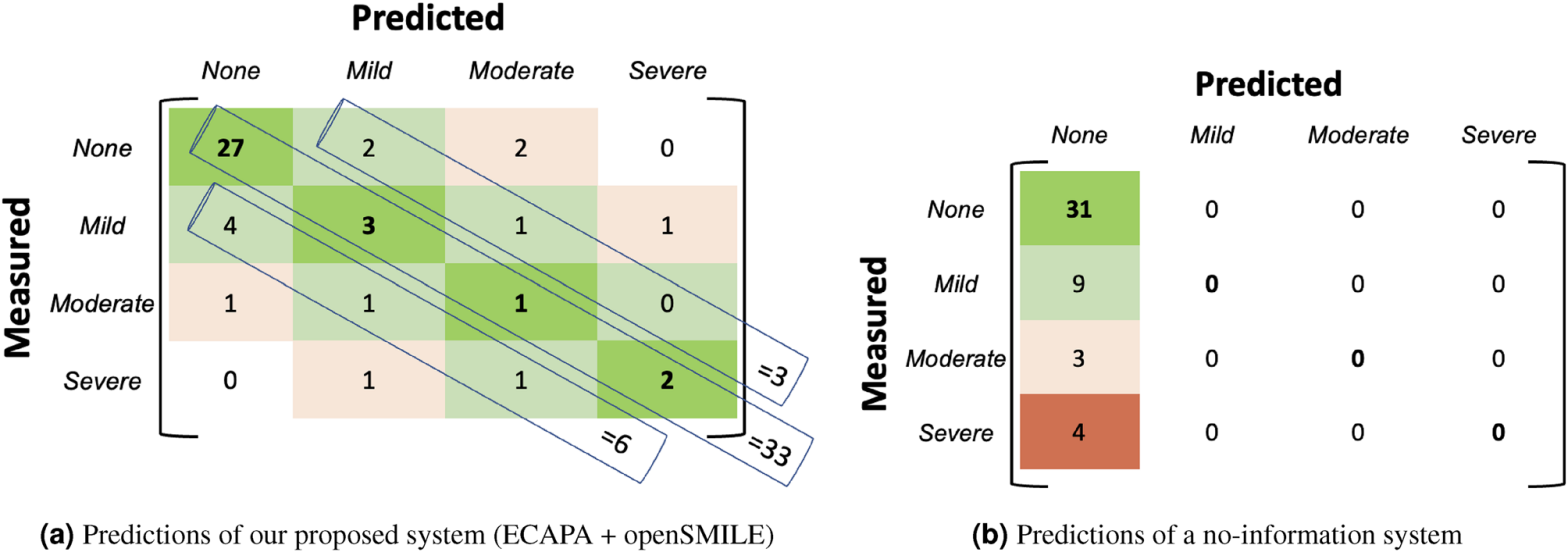
Confusion matrix obtained by considering predicted depression severity scores (PHQ-8) by (**a**) our proposed system—LSTM model trained combining ECAPA-TDNN with OpenSMILE features, and (**b**) a no-information system which predicts the mean value for every input. Fine grained clinical levels of the predicted depression severity scores obtained by dividing the depression severity scores into 4 different groups: None (PHQ-8<= 8); Mild (PHQ-8 range 9–12), Moderate (PHQ-8 range 13–16) and Severe (PHQ-8 range 17–24).

To provide context for interpreting the lower RMSE values achieved by our proposed depression assessment system (i.e. an LSTM model trained by combining ECAPA-TDNN speaker embeddings with OpenSMILE features), we present a detailed confusion matrix (see Fig. 5): We used known levels of depressive severity to evaluate the seriousness of misclassification. We found that our ECAPA-TDNN-Open SMILE model made the less severe mistakes of misclassifying between healthy controls and mild cases of depression, as shown in Fig. 5a. This compares favourably to the no-information system that is equally likely to make the bigger mistake of misclassifying severe cases of depression as controls (see Fig. 5b).

Specifically, the depression severity score values (PHQ-8) are clinically divided into 4 different groups: No depression or healthy (PHQ-8<= 8), Mild depression (PHQ-8 range 9-12), Moderate depression (PHQ-8 range 13-16) and Severe depression (PHQ-8 range 17-24). In matrix (a) on the left, we show a confusion matrix based on our system’s predicted regression scores and in matrix (b) we show a confusion matrix obtained for a Majority classifier (or a no-information system). These matrices demonstrate interesting characteristics: (1) Many of the errors made by our model are between healthy (None) and mild classes, which would likely be more tolerable, since a goal would be to track longitudinal changes; if a patient is already known to be depressed, then it may be less critical for a system to automatically detect where they lie relative to this particular border. (2) Our system misclassified only 5 patients who are clinically depressed as healthy (None), and 4 of these are mild depression cases. This is a less significant error than it would be to misclassify a severely depressed patient as being healthy (i.e. failing to flag them). The no-information system (majority predictor) classified all 16 clinically depressed patients as healthy. Indeed it would always have all of its errors in the first column: misclassifying all depressed patients as being healthy, regardless of the severity of their depression. (3) Indeed, in our system, none of the severely depressed patients are misclassified as healthy, whereas in the no-information system, 100% of severely depressed patients will be misclassified as healthy (red bin in Fig. 5b) (4) For our proposed system, most of the misclassification errors are “one bin apart” (light green diagonals in Fig. 5a), i.e. confusion between adjacent classes such as mild-none or mild-moderate, as opposed to confusion between more separated classes such as none-moderate. The no-information system misclassified all the 3 moderately depressed people as healthy and the 4 severely depressed people as healthy.

## Limitations

In this work, we showed that speaker embeddings can be used to build machine learning models for depression assessment. Using speaker embeddings in combination with acoustic features, we achieved incremental progress in performance over the previous state-of-the-art machine learning techniques for the tasks of depression severity estimation and depression detection. However, there is a need to further improve performance before deploying AI-based depression assessment systems. In this work, we considered acoustic features, but not text-based features (i.e. linguistic content). It is possible that the latter, in combination with acoustic features, might in future further improve the performance of these machine learning models. The main objective of this work is not to build machine learning models to replace human clinicians, but to develop models which can be used for measurement-based treatment and to assist (i.e. work in co-ordination with) human clinicians in making better assessment of depression. Moreover, the specificity of the current models in diagnosing depression from other mental disorders remains to be established.

## Conclusions

In this work we train a speaker embedding network on standard large datasets and then use two small clinical datasets to show that the resulting embeddings can then be used to estimate the severity of depression and to detect depression from speech. In particular, when we combine these embeddings with OpenSMILE speech features, we achieve SOTA performance on the depression severity estimation and the depression detection tasks. Further, we show that the changes in depression severity affects the speaker identification by analyzing repeated speech samples collected from a subset of speakers.

## Supplementary Information

Supplementary Information 1.

## Data Availability

Publicly available Voxceleb2 (https://www.robots.ox.ac.uk/~vgg/data/voxceleb/vox2.html) and LibriSpeech (https://www.openslr.org/12) datasets were used to train the speaker embedding models i.e., x-vector, d-vector and ECAPE-TDNN x-vector models. The DAIC-WOZ dataset is publicly available at https://dcapswoz.ict.usc.edu/). The Vocal Mind dataset generated and analyzed during the current study is not publicly available due to potential identifiable character of speech data, sensitive character of the associated information on mental disorders, and limits of consent provided by participants. The study procedures for Vocal Mind dataset, and all the experiments in this research have been carried out in accordance with the Canadian Tri-Council Policy Statement: Ethical Conduct for Research Involving Humans - TCPS 2 (2018) policy statement. The Research Ethics Board of Nova Scotia Health Authority approved all study procedures. All the participants provided written informed consent. The consent covers the publication of de-identified data and results. The consent does not permit publication of identifiable information. A proportion of participants have additionally consented for their de-identified audio recordings to be shared with other researchers in other Canadian research institutions and/or research institution outside of Canada. De-identified version of these samples are available from the corresponding author on reasonable request.
