# Supplementary material for: Manifestation of depression in speech overlaps with characteristics used to represent and recognize speaker identity
Source: Sci Rep. 2023 Jul 10;13:11155. doi: 10.1038/s41598-023-35184-7 (PMC10333314; doi:10.1038/s41598-023-35184-7)
Supplement: Supplementary file 1 — Supplementary Information 1. [file 41598_2023_35184_MOESM1_ESM.pdf]

Manifestation of Depression in Speech Overlaps with Characteristics Used to Represent and Recognize Speaker Identity

Sri Harsha Dumpala[1,2], Katerina Dikaios[3,4], Sebastian Rodriguez[1,2], Ross Langley[3], Sheri Rempel[4], Rudolf Uher[3,4], and Sageev Oore*[1,2]

[1]Dalhousie University, Faculty of Computer Science, Halifax, NS, Canada
[2]Vector Institute, Toronto, ON, Canada
[3]Dalhousie University, Psychiatry, Halifax, NS, Canada
[4]Nova Scotia Health, Halifax, NS, Canada
*sageev@dal.ca

Background

X-vectors

X-vectors are speaker embeddings that are extracted using a feed-forward deep neural network (DNN) trained to classify the N speakers in the training data[1,2]. We provide a brief description of the x-vector system which is detailed in Snyder et al[1]. The feed-forward DNN consists of layers that operate on speech frames, a statistics pooling layer that aggregates over the frame-level representations, additional layers that operate at the segment-level, and finally a softmax output layer. The non-linearities in this network are rectified linear units (ReLUs). The first 5 layers of the DNN work at the frame level, with a time-delay architecture[3]. Suppose t is the current time step. The input layer splices together frames at $\{t-2, t-1, t, t+1, t+2\}$. The second and third layers splice together the output of the previous layer at times $\{t-2, t, t+2\}$ and $\{t-3, t, t+3\}$, respectively. The next two layers, i.e., fourth and fifth layers, also operate at the frame-level, but without any added temporal context. In total, the frame level portion of the network (first 5 layers) has a temporal context of $t-8$ to $t+8$ frames. Layers vary in size, from 512 to 1536, depending on the splicing context used. The statistics pooling layer receives the output of the final frame-level layer as input, aggregates over the input segment, and computes its mean and standard deviation. These segment-level statistics are concatenated together and passed through two additional hidden layers with dimension 512 and 300 (either of these two layers may be used to compute embeddings), respectively, and finally the softmax output layer. The network is trained to classify the speakers in the training set using a negative log-likelihood loss function.

ECAPA-TDNN x-vectors

The ECAPA-TDNN[4] is an improved version of the x-vector model explained above, which achieved SOTA performance for the tasks of speaker verification and speaker diarization[5]. ECAPA-TDNN introduced several enhancements to the initial x-vector model to extract more robust speaker embeddings. Channel-dependent and context-dependent attention mechanisms are used for the pooling layer which allows the network to give higher weightage to more informative frames in the input. To increase the temporal context of the frame layers in the original x-vector system, 1-dimensional Squeeze Excitation[6] blocks are used to re-scale the channels of the intermediate frame-level feature maps to insert global context information in the locally operating convolutional blocks. 1-dimensional Res2-blocks are integrated to improve the performance while simultaneously reducing the total parameter count by using grouped convolutions in a hierarchical way. Further, Multi-layer Feature Aggregation[7] is used to merge the complementary information before the statistics pooling by concatenating the final frame-level feature map with intermediate feature maps of preceding layers. The ECAPA-TDNN network is trained for the task of classifying speakers in the train set by optimizing the additive angular margin (AAM) softmax objective function[8]. In AAM-softmax objective function, the cosine distance between the speaker embeddings is optimized.

Generalized end-to-end d-vectors

To obtain d-vector-based speaker embeddings from speech, we follow the generalized end-to-end (GE2E) speaker verification approach proposed in Wan et al[9]. We provide below a brief review of the GE2E approach below.

GE2E training is based on processing a large number of utterances at once, in the form of a batch that contains N speakers, and M utterances from each speaker. Each feature vector sequence x_{ji} ($1 \leq j \leq N$ and $1 \leq i \leq M$) represents the feature sequence (frame-level features) extracted from the ith utterance of jth speaker. These frame-level features extracted from each utterance x_{ji} were fed into a deep LSTM network with 3 LSTM layers (with 256 units each) followed by a linear layer (with

256 units). The linear layer performs an affine transformation on the last frame response of the LSTM layers. The output of the linear layer of the network is denoted as $f(x_{ji};\theta)$ where θ represents parameters of the entire neural network. The embedding vector (also known as d-vector) is defined as the $L2$ normalization of the final layer output:

$$e_{ji} = \frac{f(x_{ji},\theta)}{\|f(x_{ji},\theta)\|},$$

where e_{ji} represents the embedding vector obtained for the ith utterance of jth speaker. For each speaker in the batch is computed where the centroid c_j (which represents the voice print) of the jth speaker is obtained by computing the mean of all the embedding vectors $[e_{j1}, e_{j2}, \cdots e_{jM}]$ corresponding to j^{th} speaker. Then a similarity matrix S is computed for each batch, with $N \times M$ rows and N columns. An element $S_{ji,k}$ in the similarity matrix is defined as the scaled cosine similarity between each embeddings vector e_{ji} and all the centroids c_k ($1 \leq j,k \leq N$ and $1 \leq i \leq M$):

$$S_{ji,k} = w \cdot cos(e_{ji}, c_k) + b,$$

where w and b are learnable parameters. The model was trained using softmax on $S_{ji,k}$, which outputs 1 if $j = k$, otherwise outputs 0. The softmax loss on embeddings vector e_{ji} can be defined as:

$$L(e_{ji}) = S_{ji,j} - log \sum_{k=1}^{N} exp(S_{ji,k}).$$

This loss function enables the network to learn parameters such that embeddings vectors corresponding to j_{th} speaker are pulled close to the centroid c_j and at the same time pushed away from other centroids corresponding to other speakers.

We used the pre-trained models for extracting speaker embeddings (x-vector, ECAPA-TDNN x-vectors and d-vectors) at segment-level for each of the DAIC-WOZ and FORBOW datasets. Each segment is represented using a speaker embedding of dimension 512, 256, and 192 for x-vector, ECAPA-TDNN x-vector and d-vector, respectively. Finally, we use these speaker embeddings separately to train and test the LSTM and CNN based models for depression detection i.e., LSTM and CNN models were trained separately on x-vector, ECAPA-TDNN x-vector and d-vector speaker embeddings.

Additional Results

Depression detection using speaker embeddings: Supplementary Table S1 shows the depression assessment results obtained using different speaker embeddings i.e., x-vector, ECAPA-TDNN x-vectors and d-vector speaker embeddings. It can be observed from Supplementary Table S1 that all the three types of speaker embeddings achieve SOTA performance on depression assessment, with ECAPA-TDNN x-vectors and d-vectors achieving better performance than x-vectors.

Supplementary Table S2 shows the depression assessment results obtained by combining speaker embeddings (x-vector, ECAPA-TDNN x-vectors and d-vector) with acoustic features (COVAREP and OpenSMILE). It can be observed that the depression assessment performance improved when the speaker embeddings were combined with the acoustic features. Best performance was achieved when ECAPA-TDNN x-vectors are combined with OpenSMILE features.

			Speaker Embeddings											
			x-vector				ECAPA-TDNN				d-vector			
	Model	Context	$F_1(D)$	$F_1(H)$	BAc.	RMS	$F_1(D)$	$F_1(H)$	BAc.	RMS	$F_1(D)$	$F_1(H)$	BAc.	RMS
DAIC	DNN	1	0.29	0.71	0.51	7.30	0.31	0.75	0.54	7.18	0.32	0.74	0.53	7.09
	MK-CNN	20	0.38	0.75	0.57	6.46	0.43	0.78	0.61	6.35	0.42	0.77	0.60	6.32
	LSTM	20	0.40	0.76	0.58	6.42	**0.46**	**0.79**	**0.62**	6.31	0.44	0.78	0.61	**6.24**
VM	DNN	1	0.28	0.71	0.51	7.14	0.31	0.73	0.52	7.07	0.28	0.74	0.52	7.13
	MK-CNN	16	0.29	0.76	0.53	6.73	0.32	0.80	0.56	6.64	0.31	0.79	0.55	**6.55**
	LSTM	16	0.31	0.77	0.54	6.71	**0.34**	**0.81**	**0.58**	6.62	**0.34**	0.79	0.57	6.57

Supplementary Table S1. Performance values in terms of F_1, balanced accuracy (BAc.), and RMSE (RMS) when different speaker embeddings are used

Depression detection using demographic information: In order to understand the significance of the demographic variables such as biological sex and age in detecting depression, we trained different machine learning models (decision trees, support vector machines (SVM) and deep neural networks (DNNs)) for the task of depression detection using: (1) only biological

	COVAREP					(x-vector, COV)			(ECAPA, COV)			(d-vector, COV)		
DAIC-WOZ		$F_1(D)$	$F_1(H)$	BAc.		$F_1(D)$	$F_1(H)$	BAc.	$F_1(D)$	$F_1(H)$	BAc.	$F_1(D)$	$F_1(H)$	BAc.
	DNN	0.31	0.64	0.48	CE_d	0.31	0.72	0.52	0.32	0.75	0.54	0.32	0.74	0.53
	MK-CNN	0.35	0.70	0.52	CE_c	0.40	0.75	0.57	0.45	0.79	0.61	0.43	0.78	0.60
	LSTM	0.32	0.70	0.51	CE_l	0.40	0.77	0.59	**0.47**	**0.80**	**0.63**	0.46	0.78	0.62
	OpenSMILE					(x-vector, OS)			(ECAPA, OS)			(d-vector, OS)		
		$F_1(D)$	$F_1(H)$	BAc.		$F_1(D)$	$F_1(H)$	BAc.	$F_1(D)$	$F_1(H)$	BAc.	$F_1(D)$	$F_1(H)$	BAc.
	DNN	0.31	0.70	0.51	CE_d	0.32	0.74	0.53	0.41	0.76	0.58	0.34	0.76	0.55
	MK-CNN	0.37	0.74	0.55	CE_c	0.41	0.77	0.59	0.49	0.81	0.65	0.49	0.80	0.64
	LSTM	0.39	0.73	0.56	CE_l	0.49	0.80	0.64	**0.50**	**0.83**	**0.66**	**0.50**	**0.83**	**0.66**

	COVAREP					(x-vector, COV)			(ECAPA, COV)			(d-vector, COV)		
Vocal Mind		$F_1(D)$	$F_1(H)$	BAc.		$F_1(D)$	$F_1(H)$	BAc.	$F_1(D)$	$F_1(H)$	BAc.	$F_1(D)$	$F_1(H)$	BAc.
	DNN	0.29	0.67	0.48	CE_d	0.33	0.72	0.53	0.33	0.74	0.54	0.30	0.75	0.52
	MK-CNN	0.30	0.68	0.49	CE_c	0.33	0.77	0.51	0.34	0.80	0.57	0.34	0.79	0.56
	LSTM	0.32	0.67	0.50	CE_l	0.33	0.78	0.55	**0.37**	**0.81**	**0.60**	0.35	0.80	0.57
	OpenSMILE					(x-vector, OS)			(ECAPA, OS)			(d-vector, OS)		
		$F_1(D)$	$F_1(H)$	BAc.		$F_1(D)$	$F_1(H)$	BAc.	$F_1(D)$	$F_1(H)$	BAc.	$F_1(D)$	$F_1(H)$	BAc.
	DNN	0.24	0.72	0.48	CE_d	0.34	0.74	0.54	0.36	0.77	0.56	0.35	0.77	0.56
	MK-CNN	0.32	0.74	0.53	CE_c	0.36	0.80	0.58	0.41	0.81	0.61	0.39	0.82	0.60
	LSTM	0.34	0.75	0.54	CE_l	0.38	0.82	0.60	**0.43**	**0.84**	**0.64**	0.42	0.83	0.62

Supplementary Table S2. Depression detection performance when speaker embeddings (x-vector, ECAPA-TDNN x-vector and d-vector) are combined with COVAREP (COV) and OpenSMILE (OS) features. ECAPA refers to ECAPA-TDNN x-vector. CE_d, CE_c and CE_l refer to CE_d with DNN, MK-CNN and LSTM blocks, respectively. BAc. refers to Balanced accuracy

sex; (2) only age; and (3) both biological sex and age as inputs. Supplementary Table S3 shows the performance of different models trained using demographic variables for the task of depression detection. When only biological sex or only age was used to train the models, the models were biased towards the majority class i.e., the models were always predicting the output as healthy (and never as depressed) irrespective of the input value (Sensitivity = 0.0 and specificity = 1.0). This shows that just the gender or age might not provide sufficient information to detect depression. When both gender and age were used to train the models, models were still not able to perform depression detection nearly as well as in the case of using speaker embeddings (ECAPA-TDNN x-vectors) for depression detection. This indicates that speaker embeddings are capturing more information more than just the biological sex and age. This may also be the reason for the improved emotion classification performance using x-vector speaker embeddings[10].

Input feature	Model	$F_1(D)$	$F_1(H)$	BAc.	Sen.	Spe.	RMSE
Biological sex	Decision Tree	0.0	0.86	0.50	0.0	1.0	8.92
	SVM	0.0	0.86	0.50	0.0	1.0	8.87
	DNN	0.0	0.86	0.50	0.0	1.0	8.83
Age	Decision Tree	0.0	0.86	0.50	0.0	1.0	8.89
	SVM	0.0	0.86	0.50	0.0	1.0	8.85
	DNN	0.0	0.86	0.50	0.0	1.0	8.84
Biological sex + Age	Decision Tree	0.16	0.65	0.41	0.23	0.57	8.35
	SVM	0.12	0.64	0.37	0.22	0.56	8.47
	DNN	0.11	0.61	0.35	0.14	0.54	8.53
ECAPA-TDNN X-vector	DNN	0.31	0.73	0.52	0.32	0.75	7.07
	MK-CNN	0.32	0.80	0.56	0.32	0.81	6.64
	LSTM	**0.34**	0.81	**0.58**	**0.36**	0.80	**6.62**

Supplementary Table S3. Depression detection performance on Vocal Mind dataset when demographic variables biological sex and age were used to train machine learning models – decision trees, support vector machines (SVM) and DNNs. BAc., Sen., Spe. refer to balanced accuracy, sensitivity and specificity, respectively

To understand if there are any significant differences in age between the depressed and non-depressed classes, we compared the ages of depressed participants with that of healthy participants using Kruskall-Wallis h test. A p-value of 0.41 was obtained

showing that there are no significant differences in age between the depressed and healthy participants. This explains the low-performance of the machine learning models trained using age as input.

Model	Gender	IR	$F_1(H)$	$F_1(D)$	BAc.
LSTM	Female	≈2:1	0.78	0.50	0.64
LSTM	Male	≈3:1	0.79	0.43	0.61

(a) Gender-based depression detection on DAIC-WOZ dataset

Model	Gender	IR	$F_1(H)$	$F_1(D)$	BAc.
LSTM	Female	≈3:1	0.79	0.47	0.63
LSTM	Male	≈7:1	0.78	0.25	0.52
LSTM	Female (Under-sampled)	≈7:1	0.77	0.23	0.51

(b) Gender-based depression detection on Vocal Mind dataset

Supplementary Table S4. Gender-based depression detection performance using LSTM models with ECAPA-TDNN x-vectors as input. Female, Male refers to the model trained and tested using female and male subsets, respectively. Female (Under-sampled) is obtained by under-sampling the female subset to match the distribution of the male subset of the Vocal Mind dataset. Imbalanced ratio (IR) refers to the ratio of non-depressed to depressed samples. BAc. refers to balanced accuracy

Gender-specific depression detection: Previous works have pointed out that the non-uniform distribution of gender-based samples in terms of depressed and healthy participants, in the DAIC-WOZ dataset, led to overestimated performance of the machine leaning models[11]. This is because of the models simply learning the gender-specific information from the voice. In order to analyze contribution of the gender-agnostic information contained in speaker embeddings for depression detection, we performed gender-specific depression detection as done in previous works[12,13].

$$\begin{array}{ccc} & \overline{H} & \overline{D} \\ H & 27 & 6 \\ D & 8 & 6 \end{array}$$

(a) ECAPA

$$\begin{array}{ccc} & \overline{H} & \overline{D} \\ H & 28 & 5 \\ D & 7 & 7 \end{array}$$

(b) ECAPA + OpenSMILE

$$\begin{array}{ccc} & \overline{H} & \overline{D} \\ H & 33 & 0 \\ D & 14 & 0 \end{array}$$

(c) No information system

$$\begin{array}{ccc} & \overline{H} & \overline{D} \\ H & 66 & 15 \\ D & 14 & 8 \end{array}$$

(d) ECAPA

$$\begin{array}{ccc} & \overline{H} & \overline{D} \\ H & 68 & 13 \\ D & 12 & 10 \end{array}$$

(e) ECAPA + OpenSMILE

$$\begin{array}{ccc} & \overline{H} & \overline{D} \\ H & 81 & 0 \\ D & 22 & 0 \end{array}$$

(f) No information system

Supplementary Table S5. Confusion matrices obtained using (a–c) DAIC-WOZ dataset and (d–f) Vocal Mind dataset on one of the 5-folds. (a) using only ECAPA-TDNN features for depression detection, (b) combining ECAPA-TDNN with OpenSMILE features for depression detection and (c) no information system where the system predicts the person to be healthy (majority class) irrespective of the input, (d) using only ECAPA-TDNN features for depression detection, (e) combining ECAPA-TDNN with OpenSMILE features for depression detection and (f) no information system where the system predicts the person to be healthy (majority class) irrespective of the input. H and D refer to ground truth healthy and depressed samples, respectively. $\overline{H}$ and $\overline{D}$ refer to the predicted healthy and depressed samples, respectively

In these experiments, we divide the entire dataset into two gender-based subsets – one set with only female speakers and the other set with only male speakers. We then performed 5-fold cross validation on each subset separately. Supplementary Table S4 provides the gender-specific performance of the LSTM model with ECAPA-TDNN speaker embeddings as input.

For the DAIC-WOZ dataset (see Supplementary Table S4 (a)), both Female and Male models have similar performance with the Female model performing slightly better than the Male model. This shows that depression detection using speaker embeddings was not simply relying on gender-based information. The difference in performance between the two models may be due to the difference in imbalance ratio of non-depressed to depressed samples in each gender: for females, the imbalance ratio of non-depressed to depressed is $59 : 31 \approx 2{:}1$ whereas for males the imbalance ratio of non-depressed to depressed is $95 : 34 \approx 3{:}1$.

For the Vocal Mind dataset (see Supplementary Table S4 (b)), there is a large difference between the performance of the Female and the Male models, with the Female model performing better than the Male model. This might be accounted for by the large difference in the imbalance ratio between female participants (294:95 ≈ 3:1) and male participants (109:16 ≈ 7:1). In order to understand the effect of imbalance ratio on gender-based model performance, we under-sampled the female

samples to match that of the male i.e., randomly selected 125 female samples (109 non-depressed and 16 depressed). We then performed 5-fold cross validation on this under sampled set (Female (Under-sampled)). It can be observed that the performance of the model on the Female (under-sampled) is similar to the performance of the Male model. This shows the effect of class imbalance on the model performance.

Supplementary Table S5 shows the confusion matrices obtained using (a–c) DAIC-WOZ dataset and (d–f) Vocal Mind dataset. It can be observed that for both datasets, models trained by combining ECAPA-TDNN with OpenSMILE features better identify people with depression compared to the models trained using only ECAPA-TDNN features. Further, the no information system predicts every person to be healthy (majority class) irrespective of the input i.e., it is unable to detect people with depression.

Temporal Context in Depression Detection Supplementary Figure S1 shows the depression detection performance on the DAIC-WOZ (DAIC) and Vocal Mind (VM) datasets by considering different temporal contexts. We use two different input configurations: the first configuration uses only speaker embeddings, while the second configuration uses a combination of speaker embeddings and OpenSMILE (Spk-Emb, OS) features. Our temporal contexts range from 20 seconds (4 contiguous sub-segments) to 80 or 100 seconds (16 or 20 contiguous sub-segments). As we increase the context, the depression detection performance improves until saturation. For instance, Supplementary Figure S1 shows that for the CE_l model trained using combined speaker embeddings and OpenSMILE on the Vocal Mind dataset (i.e., VM: Spk-Emb, OS), as we increase the temporal context up to 16 segments, the performance of the CE_l model improves to an accuracy of 0.76 – indicating that the temporal relationship embodied across the segments of a speech recording provide essential cues for depression detection.

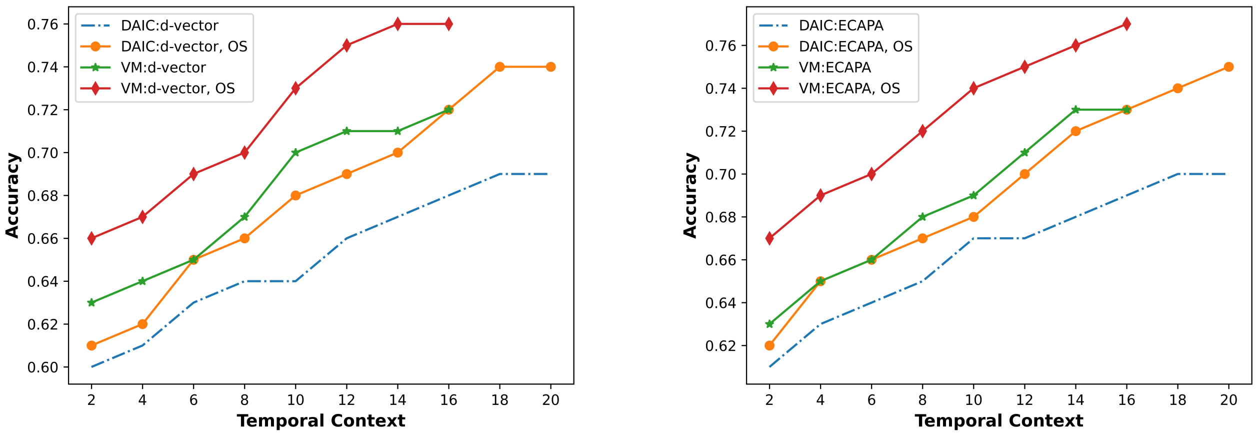

(a) Context plot for d-vector

(b) Context plot for ECAPA-TDNN x-vector

Supplementary Figure S1. Performance (Accuracy) of the LSTM (Spk-Emb) and CE_l (Spk-Emb, OS) for depression detection when the length of the context is varied from 4 up to the entire length of the shortest example in the test set, i.e. 16 and 20 respectively. VM refers to the Vocal Mind dataset

Related Work

Acoustic Representations for Depression Analysis: Several modalities such as text, speech, electronic health records, and wearable and mobile sensors were used for depression classification and severity estimation[14–17]. Speech is one such modality which attained a lot of research attention in recent times[18–20]. Depression is shown to degrade cognitive planning and psycho-motor functioning, thus affecting the human speech production mechanism[19]. These affects manifest as variations in the speech voice quality[21] and several features have been proposed to capture these variations in speech for depression analysis. Spectral features such as formants and mel-frequency cepstral coefficients (MFCCs), prosodic features such as F_0, jitter, shimmer and glottal features were initially used for depression detection[22–24]. Spectral, prosodic and other voice quality related features extracted using OpenSMILE[25] and COVAREP[26] toolkits were also used for depression analysis[27,28]. Further, features developed based on speech articulation such as vocal tract coordination features were analyzed for depression detection[21,29,30]. Recently, sentiment and emotion embeddings, representing non-verbal characteristics of speech, were used for depression severity estimation[31]. To the best of our knowledge, no other studies that we know of have explored the use

of speaker-specific information for depression detection. In this work, we consider using speaker embeddings for depression analysis.

Speaker Embeddings: Speaker embeddings refer to a low-dimensional representation of the speaker-specific characteristics that exist in the speech signal[2,32] and can be designed to be relatively *independent* of *what* the speaker is saying. Speaker representations were initially based on i-vectors, with a probabilistic linear discriminant analysis (PLDA) back-end[33]. Recently, two distinct end-to-end deep neural network based approaches were used for speaker verification, and both approaches obtained (comparable) SOTA performance[2,9]. In Snyder et al., speaker embeddings, also referred to as x-vectors, were extracted from a time-delay deep neural network (TDNN) trained for the task of speaker verification[2]. In contrast, speaker embeddings, also referred to as d-vectors, were extracted from an end-to-end LSTM network trained for speaker verification[9]. Subsequent improvements to the TDNN architecture of x-vectors have further improved the performance of speaker verification[4,34]. In Emphasized Channel Attention, Propagation and Aggregation TDNN (ECAPA-TDNN[4]), the TDNN architecture was enhanced for the task of speaker classification by introducing improvements related to channel attention, propagation and aggregation. In this work, we analyze three different variants of speaker embeddings i.e., x-vectors[2], d-vectors[9] and ECAPA-TDNN x-vectors[4].

Deep learning for depression diagnosis: Recently, the application of deep learning techniques have significantly boosted the performance of depression detection using speech[28,29,35–37]. Initially deep neural networks (DNNs) with fully-connected layers were trained for depression detection[35]. Later, convolutional neural networks (CNNs) and recurrent neural networks with long short-term memory (LSTM) units were shown to achieve better performance on depression detection[28,37]. Recently, CNN-LSTM and dilated CNNs were used for depression detection from speech to achieve SOTA performance[29,36]. In this work, we use speaker embeddings to train multi-kernel CNNs[38], and LSTM models for depression analysis.

Temporal Context in Depression Detection: A few studies have analyzed the significance of the total duration of the audio recording on the depression detection performance[39–41]. These works have shown that longer the duration, better the performance. In Yang et al. and Pampouchidou et al., the analysis was performed by considering multiple modalities i.e, audio, visual and text[39,40], whereas in Rutowski et al., automatic speech-to-text transcriptions were used to analyze the effect of duration on depression detection performance[41]. In this work, we use the acoustic features extracted from speech to analyze the effect of varying the number of contiguous speech segments on the performance of LSTM and CNN models trained for depression detection.

References

1. Snyder, D., Garcia-Romero, D., Povey, D. & Khudanpur, S. Deep neural network embeddings for text-independent speaker verification. In *Interspeech*, 999–1003 (2017).

2. Snyder, D., Garcia-Romero, D., Sell, G., Povey, D. & Khudanpur, S. X-vectors: Robust dnn embeddings for speaker recognition. In *ICASSP*, 5329–5333 (IEEE, 2018).

3. Peddinti, V., Povey, D. & Khudanpur, S. A time delay neural network architecture for efficient modeling of long temporal contexts. In *Sixteenth annual conference of the international speech communication association* (2015).

4. Desplanques, B., Thienpondt, J. & Demuynck, K. Ecapa-tdnn: Emphasized channel attention, propagation and aggregation in tdnn based speaker verification. *arXiv preprint arXiv:2005.07143* (2020).

5. Dawalatabad, N. *et al.* Ecapa-tdnn embeddings for speaker diarization. *arXiv preprint arXiv:2104.01466* (2021).

6. Hu, J., Shen, L. & Sun, G. Squeeze-and-excitation networks. In *Proceedings of the IEEE conference on computer vision and pattern recognition*, 7132–7141 (2018).

7. Gao, Z. *et al.* Improving aggregation and loss function for better embedding learning in end-to-end speaker verification system. In *INTERSPEECH*, 361–365 (2019).

8. Deng, J., Guo, J., Xue, N. & Zafeiriou, S. Arcface: Additive angular margin loss for deep face recognition. In *Proceedings of the IEEE/CVF Conference on Computer Vision and Pattern Recognition*, 4690–4699 (2019).

9. Wan, L., Wang, Q., Papir, A. & Moreno, I. L. Generalized end-to-end loss for speaker verification. In *2018 IEEE International Conference on Acoustics, Speech and Signal Processing (ICASSP)*, 4879–4883 (IEEE, 2018).

10. Pappagari, R., Wang, T., Villalba, J., Chen, N. & Dehak, N. x-vectors meet emotions: A study on dependencies between emotion and speaker recognition. In *ICASSP* (IEEE, 2020).

11. Bailey, A. & Plumbley, M. D. Gender bias in depression detection using audio features. In *2021 29th European Signal Processing Conference (EUSIPCO)*, 596–600 (IEEE, 2021).

12. Cummins, N., Vlasenko, B., Sagha, H. & Schuller, B. Enhancing speech-based depression detection through gender dependent vowel-level formant features. In *Conference on artificial intelligence in medicine in Europe*, 209–214 (Springer, 2017).

13. Vlasenko, B., Sagha, H., Cummins, N. & Schuller, B. Implementing gender-dependent vowel-level analysis for boosting speech-based depression recognition. In *Interspeech* (2017).

14. Yadav, S. *et al.* Identifying depressive symptoms from tweets: Figurative language enabled multitask learning framework. In *Proceedings of the 28th International Conference on Computational Linguistics*, 696–709 (2020).

15. Farruque, N., Huang, C., Zaiane, O. & Goebel, R. Basic and depression specific emotion identification in tweets: multi-label classification experiments. *arXiv preprint arXiv:2105.12364* (2021).

16. Pedrelli, P. *et al.* Monitoring changes in depression severity using wearable and mobile sensors. *Front. psychiatry* **11**, 584711 (2020).

17. Pellegrini, A. M. *et al.* Estimating longitudinal depressive symptoms from smartphone data in a transdiagnostic cohort. *Brain behavior* **12**, e02077 (2022).

18. Quatieri, T. F. & Malyska, N. Vocal-source biomarkers for depression: A link to psychomotor activity. In *Interspeech* (2012).

19. Cummins, N. *et al.* A review of depression and suicide risk assessment using speech analysis. *Speech communication* **71**, 10–49 (2015).

20. Slavich, G. M., Taylor, S. & Picard, R. W. Stress measurement using speech: Recent advancements, validation issues, and ethical and privacy considerations. *Stress* **22**, 408–413 (2019).

21. Williamson, J. R., Quatieri, T. F., Helfer, B. S., Ciccarelli, G. & Mehta, D. D. Vocal and facial biomarkers of depression based on motor incoordination and timing. In *Proceedings of the 4th International Workshop on Audio/Visual Emotion Challenge* (2014).

22. Low, L. A., Maddage, N. C., Lech, M., Sheeber, L. & Allen, N. Influence of acoustic low-level descriptors in the detection of clinical depression in adolescents. In *ICASSP* (IEEE, 2010).

23. Cummins, N., Epps, J., Breakspear, M. & Goecke, R. An investigation of depressed speech detection: Features and normalization. In *Interspeech* (2011).

24. Simantiraki, O., Charonyktakis, P., Pampouchidou, A., Tsiknakis, M. & Cooke, M. Glottal source features for automatic speech-based depression assessment. In *INTERSPEECH*, 2700–2704 (2017).

25. Eyben, F., Wöllmer, M. & Schuller, B. Opensmile: the munich versatile and fast open-source audio feature extractor. In *Proc. ACM conference on Multimedia*, 1459–1462 (2010).

26. Degottex, G., Kane, J., Drugman, T., Raitio, T. & Scherer, S. Covarep—a collaborative voice analysis repository for speech technologies. In *ICASSP*, 960–964 (IEEE, 2014).

27. Valstar, M. *et al.* Avec 2016: Depression, mood, and emotion recognition workshop and challenge. In *Proc. ACM workshop on Audio/visual emotion challenge*, 3–10 (2016).

28. Al Hanai, T., Ghassemi, M. M. & Glass, J. R. Detecting depression with audio/text sequence modeling of interviews. In *Interspeech*, 1716–1720 (2018).

29. Huang, Z., Epps, J. & Joachim, D. Exploiting vocal tract coordination using dilated cnns for depression detection in naturalistic environments. In *ICASSP*, 6549–6553 (IEEE, 2020).

30. Seneviratne, N., Williamson, J. R., Lammert, A. C., Quatieri, T. F. & Espy-Wilson, C. Extended study on the use of vocal tract variables to quantify neuromotor coordination in depression. In *Proc. Interspeech*, vol. 2020 (2020).

31. Dumpala, S. H. *et al.* Estimating severity of depression from acoustic features and embeddings of natural speech. In *ICASSP 2021-2021 IEEE International Conference on Acoustics, Speech and Signal Processing (ICASSP)*, 7278–7282 (IEEE, 2021).

32. Snyder, D. *et al.* Deep neural network-based speaker embeddings for end-to-end speaker verification. In *SLT Workshop*, 165–170 (IEEE, 2016).

33. Dehak, N., Kenny, P. J., Dehak, R., Dumouchel, P. & Ouellet, P. Front-end factor analysis for speaker verification. *IEEE Transactions on Audio, Speech, Lang. Process.* **19**, 788–798 (2010).

34. Snyder, D. *et al.* Speaker recognition for multi-speaker conversations using x-vectors. In *ICASSP 2019-2019 IEEE International Conference on Acoustics, Speech and Signal Processing (ICASSP)*, 5796–5800 (IEEE, 2019).

35. Tasnim, M. & Stroulia, E. Detecting depression from voice. In *Canadian Conference on Artificial Intelligence*, 472–478 (Springer, 2019).

36. Ma, X., Yang, H., Chen, Q., Huang, D. & Wang, Y. Depaudionet: An efficient deep model for audio based depression classification. In *workshop on Audio/visual emotion challenge* (2016).

37. Chlasta, K., Wołk, K. & Krejtz, I. Automated speech-based screening of depression using deep convolutional neural networks. *Procedia Comput. Sci.* **164**, 618–628 (2019).

38. Sheikh, I., Dumpala, S. H., Chakraborty, R. & Kopparapu, S. K. Sentiment analysis using imperfect views from spoken language and acoustic modalities. In *Proc. Grand Challenge and Workshop on Human Multimodal Language*, 35–39 (2018).

39. Yang, L. *et al.* Decision tree based depression classification from audio video and language information. In *Proc. ACM workshop on Audio/visual emotion challenge*, 89–96 (2016).

40. Pampouchidou, A., Simantiraki, O., Fazlollahi, A., Pediaditis, M. *et al.* Depression assessment by fusing high and low level features from audio, video, and text. In *Proc. ACM workshop on Audio/visual emotion challenge*, 27–34 (2016).

41. Rutowski, T., Harati, A., Lu, Y. & Shriberg, E. Optimizing speech-input length for speaker-independent depression classification. In *INTERSPEECH*, 3023–3027 (2019).
